# Hyperendemicity and genotype diversity of hepatitis B virus among patients attending the University of Abuja Teaching Hospital, Nigeria

**DOI:** 10.3205/dgkh000566

**Published:** 2025-07-09

**Authors:** Chinwe Ndidi Ugwu, Amos Dangana, Hassan Suleiman Chunta, Ishaku Akyala Adamu, Nanpon Miri, Mangpin Leviticus Dansura, Bwede Eugene Samuel, Felix Villeng Gagari, Johnson Adeyemi Ojo, Nyiri Miriam Gyang, Nkiruka Lynda Uzoebo, Helen Daniel Nanbol, Gabriel Bernice Nyinishu, Aisha Daminso Barde, Idris Nasir Abdullahi

**Affiliations:** 1 Biorepository Laboratory, Nigeria Centre for Disease Control and Prevention, Abuja, Nigeria; 2 National Reference Laboratory, Nigeria Center for Disease Control and Prevention, Abuja, Nigeria; 3 Global Health and Infectious Diseases Control Institute, Nasarawa State University, Keffi, Nigeria; 4 Mega Laboratory, Nigeria Center for Disease Control and Prevention, Abuja, Nigeria; 5 School of Medical Laboratory Science, Plateau State College of Health Technology, Pankshin, Nigeria; 6 Sherrif Integrated Healthcare Limited, Abuja, Nigeria; 7 Department of Medical Laboratory Science, Faculty of Allied Health Sciences, College of Medical Sciences, Ahmadu Bello University, Zaria, Nigeria

**Keywords:** HBsAg, HBV genotype, endemicity, Nigeria

## Abstract

**Background:**

Hepatitis B virus (HBV) infection remains a significant global public health issue, particularly in sub-Saharan Africa, due to inadequate healthcare and research into its genetic epidemiology. This study aims to determine the frequency of HBV antigens, antibodies, and genotypes among febrile patients attending the University of Abuja Teaching Hospital (UATH), Nigeria.

**Methods:**

This cross-sectional study enrolled 100 patients, whose blood samples were collected and screened for HBV surface antigen (HBsAg) and four other structural antigens and antibodies by Lateral Flow Assay. All HBsAg-positive samples were genotyped using type-specific polymerase chain reactions. Structured questionnaires were used to collate the sociodemographic variables of the patients.

**Results:**

HBsAg-seropositivity was 31%. The distribution of HBV genotypes was as follows: genotype E was predominant (22.6%), followed by genotype B (16.1%). Of the HBsAg-positive individuals, all were HBsAb-seronegative, 3.2%, 74.2%, and 90.3% were HBeAg, HbeAb, and HBcAb seropositive, respectively. Genotypes B, C, and D were detected in 16.1%, 3.2%, and 3.2%, respectively. Based on the number of HBV genotypes per individual, 9.7% had a single genotype, 16.1% had double genotypes, and 74.2% had triple genotypes. Higher educational qualification was significantly associated with triple HBV genotypes per individual (*p*=0.04).

**Conclusion:**

Very high seroprevalence of HBV was found and genotype E predominated. The presence of within-host multiple HBV genotypes was identified for the first time in Nigeria. This indicates the genetic heterogeneity of HBV in northern Nigeria and suggests potential effects on the control measures available.

## Zusammenfassung

**Einleitung:**

Die Infektion mit dem Hepatitis-B-Virus (HBV) ist nach wie vor ein weltweites Problem der öffentlichen Gesundheit, insbesondere in den afrikanischen Ländern südlich der Sahara, da die medizinische Versorgung und die Erforschung der genetischen Epidemiologie unzureichend sind. Ziel dieser Studie ist es, die Häufigkeit von HBV-Antigenen, -Antikörpern und -Genotypen bei fiebrigen Patienten zu bestimmen, die das Lehrkrankenhaus der Universität Abuja (UATH), Nigeria, besuchen.

**Methode:**

An der Querschnittsstudie nahmen 100 Patienten teil, deren Blutproben entnommen und mittels Lateral Flow Assay auf HBV-Oberflächenantigen (HBsAg) und vier weitere strukturelle Antigene und Antikörper untersucht wurden. Alle HBsAg-positiven Proben wurden mittels typspezifischer PCR genotypisiert. Mit strukturierten Fragebögen wurden die soziodemografischen Variablen der Patienten erfasst.

**Ergebnisse:**

Die HBsAg-Seropositivität betrug 31%. Die Verteilung der HBV-Genotypen war wie folgt: Der Genotyp E war vorherrschend (22,6%), gefolgt vom Genotyp B (16,1%). Von den HBsAg-positiven Personen waren alle HBsAb-seronegativ, 3,2%, 74,2% und 90,3% waren HBeAg-, HBeAb- bzw. HBcAb-seropositiv. Die Genotypen B, C und D wurden bei 16,1%, 3,2% bzw. 3,2% nachgewiesen. Gemessen an der Anzahl der HBV-Genotypen pro Person hatten 9,7% einen Genotyp, 16,1% hatten zwei Genotypen und 74,2% hatten drei Genotypen. Der Bildungsabschluss war signifikant mit der Anzahl der dreifachen HBV-Genotypen pro Person verbunden (*p*=0,04).

**Schlussfolgerung:**

Es wurde eine sehr hohe Seroprävalenz von HBV festgestellt, wobei der Genotyp E vorherrschte. Das Vorhandensein mehrerer HBV-Genotypen innerhalb eines Wirts wurde zum ersten Mal in Nigeria festgestellt. Das weist auf die genetische Heterogenität von HBV in Nordnigeria hin mit möglichen Auswirkungen auf die verfügbaren Kontrollmaßnahmen.

## Introduction

Hepatitis B virus (HBV) infection is a major global health concern, causing significantly higher morbidity and mortality in developing countries [1]. In Nigeria, HBV is reported to be the most common cause of liver disease [2]. Individuals who test HBsAg-seropositive need to have other hepatitis B markers (anti-HBs, HBeAg, anti-HBe, and HBV DNA) evaluated to determine the stage and severity of infection [3].

HBV is endemic in Nigeria [4]. However, there is limited data on the comprehensive evaluation of hepatitis B markers to provide critical data to optimize and assess the impact of current prevention and control strategies, including disease surveillance and diagnoses, vaccination policies, and management [1]. Nigeria has a very high risk of HBV infection because of the country’s poor immunization rates and the fact that up to 75% of the population will be exposed to the virus at some point in their life [5].

Before the characterization of HBV genotypes, strains were distinguished by serological analysis, based on the immunoreactivity of an antibody to a limited number of amino acids in the major surface antigen, HBsAg [6]. Subsequently, HBV now exists in 10 known genotypes (A–J) and 40 subgenotypes based on an intergroup divergence of 8% or 4%, using the gene-S sequence [7]. The natural course of an HBV infection may be influenced by the genotypes or subgenotypes of the virus, but the identification of a particular genotype or subgenotype is not necessary for the administration of antiviral medication [8]. However, given that the genotypes are known to differ across geographic regions and have a high correlation with ethnicity, they have been proposed as valuable epidemiological identifiers [9].

Acute self-limiting hepatitis is linked to genotype D, chronic active hepatitis is linked to genotype A, and liver cancer is linked to genotype B [8], [9]. Despite being widespread, genotype A is mostly found in Northern Europe, Central Africa, and North America [7], [10]. Southeast Asia and the Far East have reported cases of HBV infections due to genotypes B and C [11]. Genotype D is mostly found in the Mediterranean area, but is distributed throughout the world [11]. 

West Africans and Native Americans primarily have genotypes E and F, respectively. Lately, genotype H has been discovered in Central America, and genotype G has been discovered in the United States and France [12]. Genotypes I and J are understudied. However, Asian genotypes I and J are thought to be the product of recombination events with other genotypes [12]. 

If HBsAg persists for more than 6 months, spontaneous clearance is very improbable, and the infected individual is a chronic HBV carrier [13]. However, during the acute phase of infection, following HBsAg detection, other viral markers can be easily detected, including DNA polymerase and HBeAg [13]. HBeAg appears shortly after the appearance of HBsAg and disappears within several weeks as acute hepatitis resolves [14]. Its presence in the serum correlates with the presence of viral replication in the liver and HBsAg detection, while its disappearance, associated with anti-HBe detection, is considered a sign of the absence of viral replication and spontaneous resolution of acute infection [14]. Anti-HBc IgM antibodies are detectable at the outset of clinical disease; as the infection evolves, IgM anti-HBc levels gradually decline, often becoming undetectable within 6 months, and IgG class predominates, remaining for a long period (sometimes life-long) at detectable levels [15]. IgG anti-HBc is correlated with prior infection, whereas IgM anti-HBc often suggests recent or ongoing HBV replication [16]. In cognizance of the enormous clinic-epidemiological value of characterizing the serological markers and genotypes in HBV-infected individuals, this study aims to determine the frequency of HBV antigens, antibodies, and genotypes among febrile patients attending the University of Abuja Teaching Hospital, Nigeria. 

## Materials and methods

### Study center

This cross-sectional study was performed at the University of Abuja Teaching Hospital (UATH) Gwagwalada Municipal Area in the Federal Capital Territory (Abuja), Nigeria. 

### Study size and participants

A minimum sample size of 89 was calculated from the 6% seroprevalence of HBsAg previously reported in the same study area [17]. All patients who consented to participate were randomly enrolled at the various departments and clinics of the University of Abuja Teaching Hospital. Pregnant women, diabetic patients, and people living with HIV and AIDS at the time of sample collection were excluded. Parents provided informed consent on behalf of the children.

### Ethics approval

The study was approved (No.: FCT/UATH/HREC/14723) by the Human Research Ethics Committee (HREC) of the University of Abuja Teaching Hospital Gwagwalada (UATH), Abuja, Nigeria. All authors gave informed consent before enrolment into the study. Sample collection and data collation from the study participants were according to the Declaration of Helsinki.

### Sample collection and processing

Each participant had a total of five milliliters (5 mL) of whole blood drawn and separated into 3 mL in an EDTA tube and 2 mL in a plain tube. To extract gDNA, 2 ml (microliters: 200 µL) of plasma were extracted directly from the EDTA-anticoagulated blood. The EDTA-anticoagulated blood was centrifuged at 5,000 revolution per minute for five minutes to remove the plasma and separate it from the red blood cells. After that, the transparent plasma layer was placed in cryovials and kept cold until analysis. Furthermore, the sera were extracted from the clotted whole blood in the plain tube. The sera were used for HBV serological tests, while the plasma samples were used for HBV DNA testing by PCR at the National Reference Laboratory (NRL), Nigeria Center for Disease and Prevention. 

### Laboratory analytical procedures

Detection of HBV serologic markers (HBsAg, HBsAb, HBeAg, HBeAb and HBcAb) was performed using the HBV-5 panel test kit (Abbott Laboratories Biotech Co. Ltd) was used. The test was based on (a) the direct antigen-antibody-antigen “Sandwich” method for HBsAg, HBsAb, HBcAg and HbeAg, and (b) the indirect antigen-antibody-antibody assay for HBcAb. The sandwich immunochromatographic strips method contains gold nanoparticles pre-coated with monoclonal antibodies specific to HBsAg, HBeAg, HBc IgM, or antigen specific to HBs recombinant monoclonal antibodies detectable in serum samples. The test was conducted and the result interpretedout according to the manufacturer’s instructions. 

The HBV DNA was extracted from the plasma of HBsAg-positive patients using a Mini Kit (Qiagen, Hilden, Germany) following the manufacturer’s recommendations. The HBV genotypes were detected using the nested PCR using the primers (Table 1 (Tab. 1)).

Nested Multiplex PCR was used for HBV DNA amplification using the universal primers (P1 and S1–2) for the outer primers and two different mixtures containing type-specific inner primers. The first PCR was carried out in a final reaction volume of 25 µl containing 2.5 µl of 1XPCR buffer, 1.0 µl of MgCl_2_, 0.8µl of forward primer, 0.8 µl of reverse primer, 0.8 µl of dNTPs, 0.2 µl of Taq, 13.0 µl of PCR water, and 5.0 µl of DNA. The thermocycler (Bonn-Bad Godesberg, Germany) was programmed to first incubate the samples for 10 min at 95°C, followed by 40 cycles of 94°C for 20s, 55°C for 20s, and 72°C for 1 min. Two second-round PCRs were performed for each sample, with the common universal sense primer (B2) and mix A for types A through C, and the common universal antisense primer (B2R) and mix B for types D through F. A 1-µl aliquot of the first PCR product was added into two tubes containing the second sets of each of the inner primer pairs, each of the deoxynucleotides, Taq and PCR buffer, as in the first reaction. These were amplified for 40 cycles, incubating at 95°C for 10 min, 20 cycles of amplification at 94°C for 20 s, 58°C for 20 s, 72°C for 30 s, an additional 20 cycles of 94°C for 20 s, 60°C for 20 s, and 72°C for 30 s. Genotype-specific DNA bands were used to identify the HBV genotypes.

The DNA from the PCR products was separated on a 10% agarose gel, stained with SYBR green, and visualized under ultraviolet light. 

## Results

Out of the 100 patients tested, 31% were HBsAg-positive. Among the 31 HBsAg-positive individuals, most of them were ≤30 years of age (51.6%), 51.6% were females, and 48.4% were married (Table 2 (Tab. 2)). Regarding educational qualifications, a majority had tertiary education (67.7%). Most of the participants (90.3%) reported no alcohol consumption. Regarding medical history, 71.0% had no prior hepatitis B history. Notably, none of the participants (100%) had been vaccinated against HBV. Various forms of chronic comorbidity were reported in 48.4% of the participants (Table 2 (Tab. 2)). 

All 31 HBsAg-positive individuals tested negative for HBsAb (100%). For HBeAg, 3.2% tested positive. Moreover, 74.2% tested positive for HBeAb, and 90.3% tested positive for HBcAb (Table 3 (Tab. 3)).

Of the 31 HBV-infected participants, HBV genotype E was predominant (22.6%). Genotypes B, C, and D were detected in 16.1%, 3.2%, and 3.2%, respectively. However, genotypes A and F were not detected at all (100.0%) (Table 4 (Tab. 4)). Concerning the number of HBV genotypes in an individual, 9.7% had a single genotype, 16.1% had double genotypes, and 74.2%, had triple genotypes. 

All participants within the age group ≥41 years had the highest percentage of triple HBV genotypes, followed by those ≤30 years (75%) (*p*=0.16). Both married and single individuals exhibit similar patterns in the number of HBV genotypes (*p*=0.92). Males had relatively higher (80%) triple HV genotypes than did females (68.8%) (*p*=0.47).

Educational qualification was significantly associated with multiple HBV genotypes per individual (*p*=0.04). In this regard, individuals with a secondary education had the highest frequency of triple HBV genotypes (48.4%) (Table 5 (Tab. 5)).

## Discussion

In any given population, an HBsAg prevalence estimate of more than 8% is deemed high [18]. The high HBsAg rate in this study indicates that Nigeria is HBV hyperendemic [19]. Globally, the prevalence of chronic HBV infection varies, from less than 1% in low-endemicity areas to more than 30% in highly endemic areas, depending on sociodemographic factors, lifestyle, clinical conditions, and vaccination coverage [20]. Similar high HBV seroprevalence in Nigeria has previously been reported in 44.7% of healthy pupils, 32% of patients, 29.7% in chronically infected persons, and 51.9% in people living with HIV/AIDS [21], [22], [23], [24], [25]. It is important to remark that most cross-sectional studies reported HBsAg frequency ranges of 4–22% in Nigeria [1]. 

In a global meta-analysis of the prevalence of HBV, it was reported that most developing countries in sub-Saharan Africa and Southeast Asia are hyperendemic for HBV infection with range of 10-30% [26]. 

All participants in the present study were negative for HBsAb, indicating a lack of immunity from prior infection or vaccination. Hence, they all had active hepatitis B. Of these, only one individual was HBeAg, a marker of active replication in the liver cells. This implies that the individual could be quite contagious due to their potentially high viral loads [27]. HBeAb, an indicator of resolved infection or low replication, was identified in most HBsAg-seropositive individuals. 

The predominance of HBV genotype E in the present study conforms with most previous reports in Nigeria in different subpopulations with varying clinical conditions [21], [28], [29], [30]. This indicates the establishment of HBV genotype E in Nigeria. Specifically, genotype E is prevalent in West and Central Africa [11]. 

When many HBV genotypes are present in the same host, it is referred to as an HBV mixture. This can happen as a result of super- or co-infection [31]. In the present study, many within-host dual or triple HBV genotypes were identified. These are due to clusters associated with multiple introductions into the studied populations. When two or more different HBV genotypes or sub-genotypes infect the same host cell, they exchange genetic material during replication, a process known as recombination [32]. This phenomenon could have an impact on clinical outcomes, such as the development of liver fibrosis, cirrhosis, and hepatocellular cancer [31]. To our knowledge, this is the first report of within-host multiple HBV genotypes in Nigeria. Similar findings were recently reported in individual pregnant women in Ghana [33]. The absence of genotypes A and F is consistent with their rarity outside specific geographic regions. 

Very high seroprevalence of HBV was found and genotype E predominated. Multiple HBV genotypes per individual were identified for the first time in northern Nigeria. This underscores the genetic heterogeneity of the virus in this region and suggests potential implications for disease progression, treatment response, and vaccine efficacy. It is recommended to perform phylogenomic analyses of the predominant HBV genotypes E and B identified in the study center to understand the evolutionary trajectory within and outside the region. Although the sample size of this study was statistically adequate, larger cohorts are required to improve epidemiological relevance. 

In conclusion, there is a need to maintain HBV vaccination programs in infancy and revaccinate individuals at high risk of infection in adulthood. Moreover, other preventive measures, such as safe sex practices and avoidance of sharing syringe needles and sharp objects, should be encouraged. 

## Notes

### Ethical approval 

This study was approved (No.: FCT/UATH/HREC/14723) by the Human Research Ethics Committee (HREC) of the University of Abuja Teaching Hospital Gwagwalada (UATH), Abuja, Nigeria.

### Authors’ ORCIDs

- Abdullahi IN: https://orcid.org/0000-0002-5511-1272
- Dangana A: https://orcid.org/0000-0001-9955-3841
- Ugwu CN: https://orcid.org/0009-0008-6910-8665
- Akyala AI: https://orcid.org/0000-0002-4168-6104
- Dansura ML: https://orcid.org/0009-0008-1965-5046
- Samuel BE: https://orcid.org/0009-0007-8690-4850
- Gagari VF: https://orcid.org/0009-0003-0460-3888
- Gyang NM: https://orcid.org/0009-0002-0560-8560
- Uzoebo NK: https://orcid.org/0009-0000-8001-1444

### Competing interests

The authors declare that they have no competing interests.

## Figures and Tables

**Table 1:**
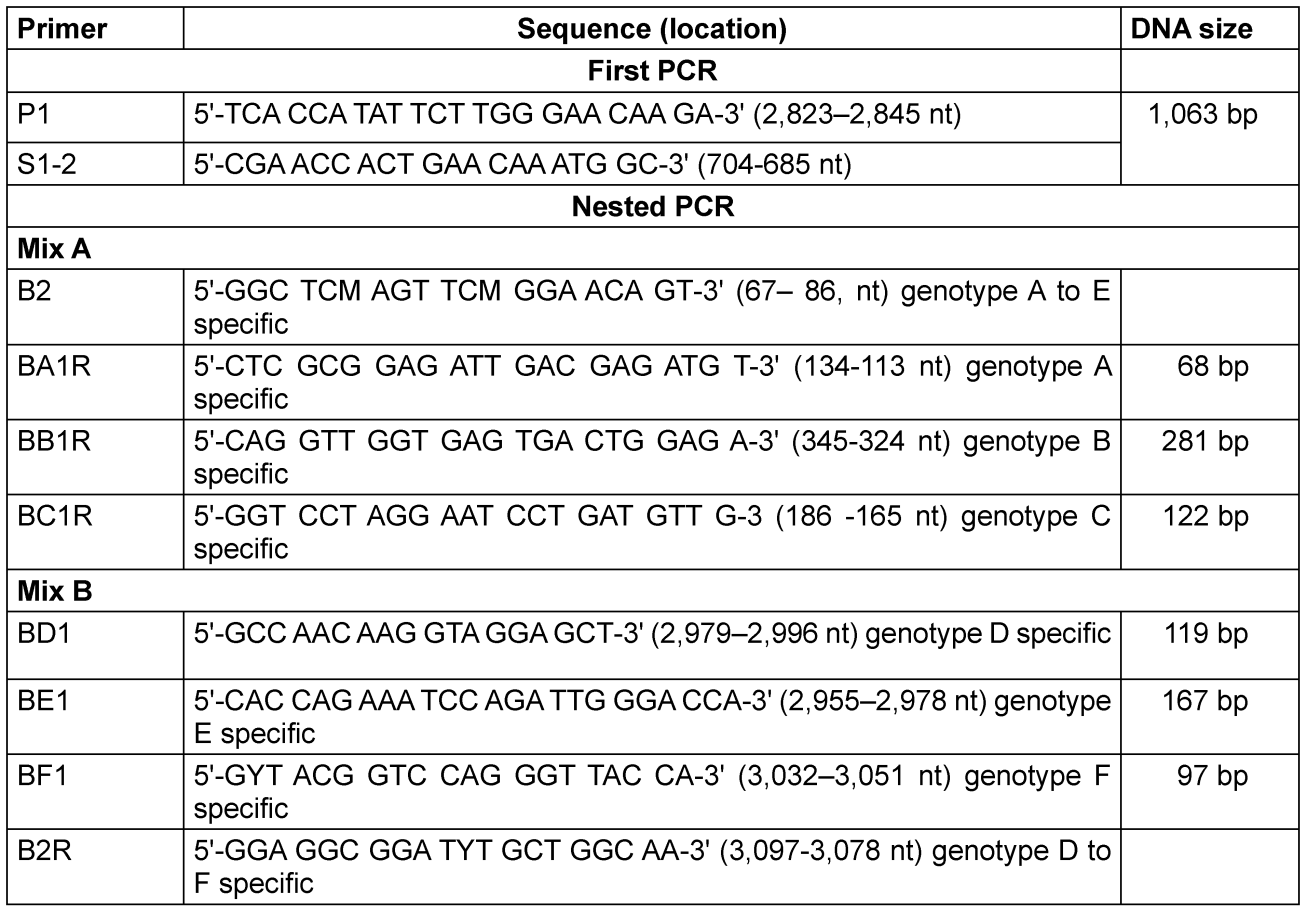
Primers for HBV genotyping (adopted from Naito et al. [34])

**Table 2:**
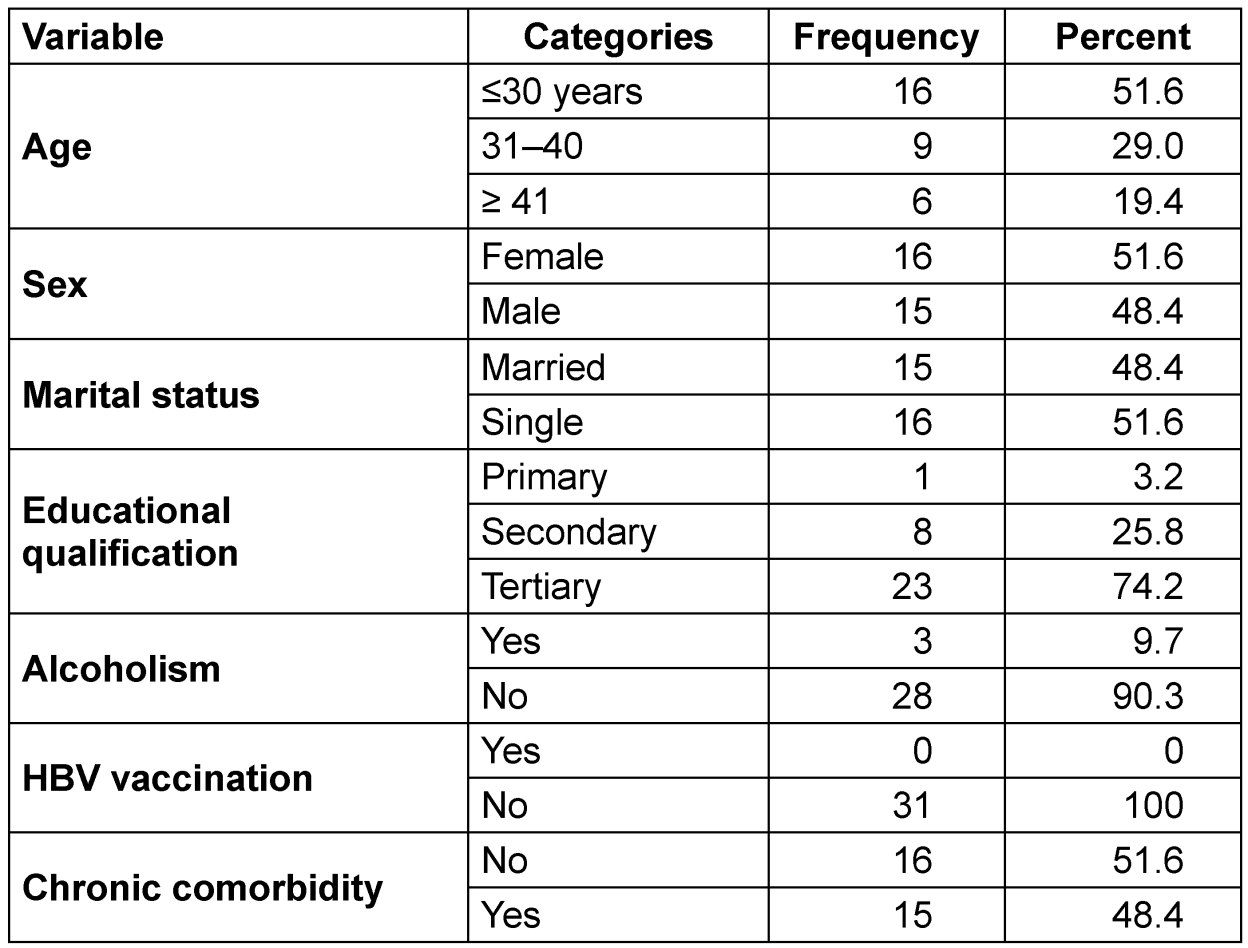
Demographic and risk factors in HBsAg-positive participants

**Table 3:**
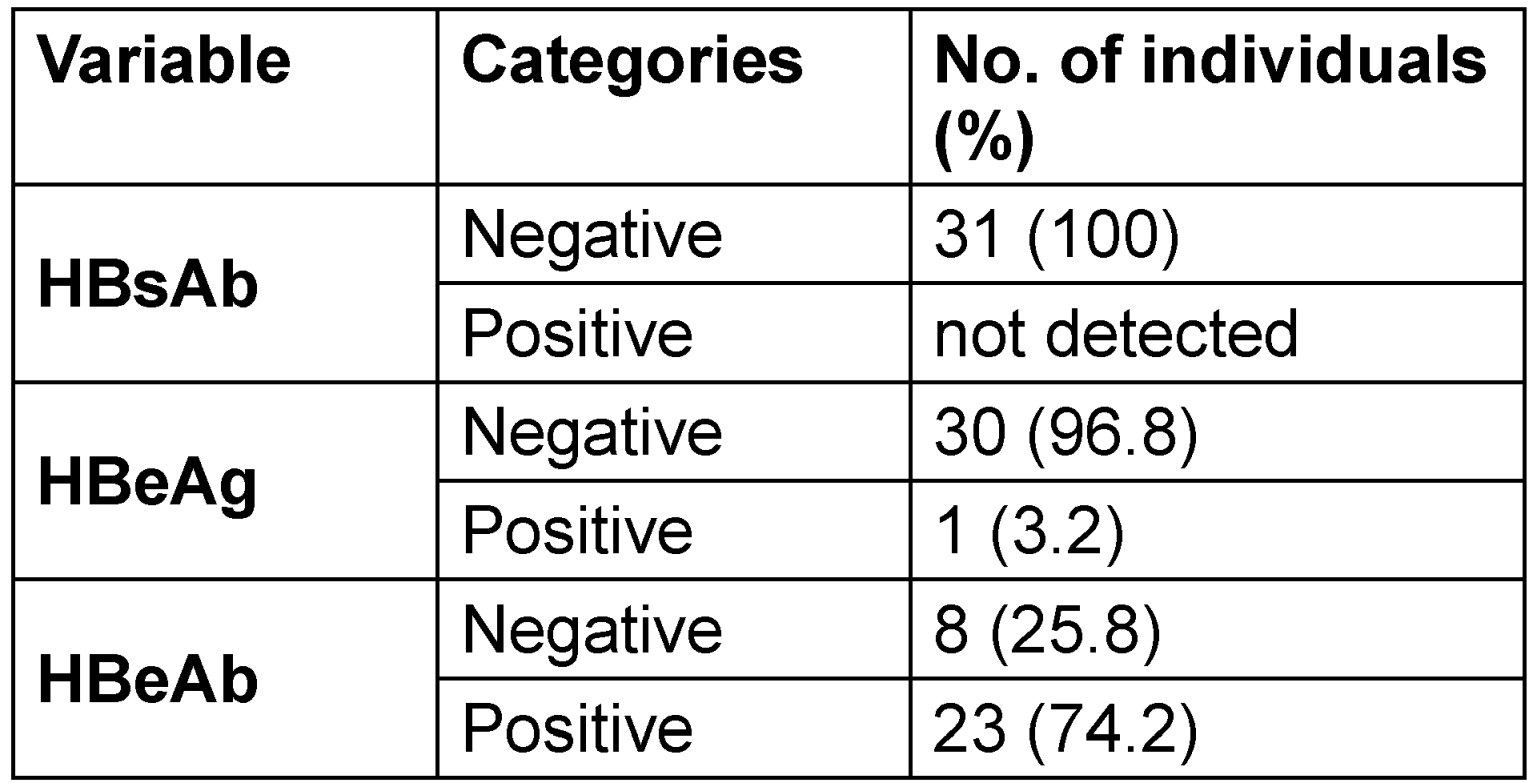
Hepatitis B serological markers in the HBsAg positive individuals

**Table 4:**
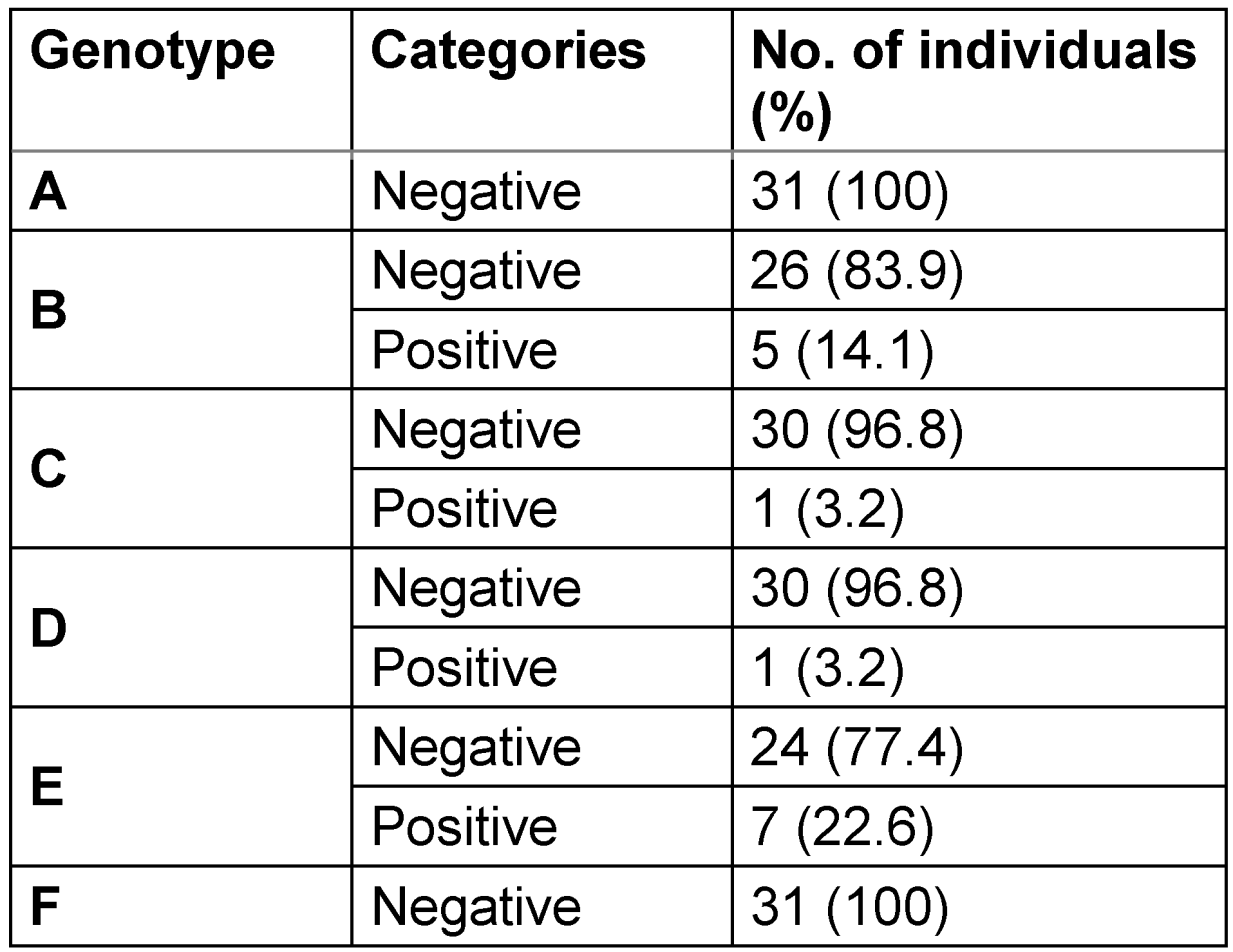
Frequency of Hepatitis B virus genotypes among HbsAg-positive individuals

**Table 5:**
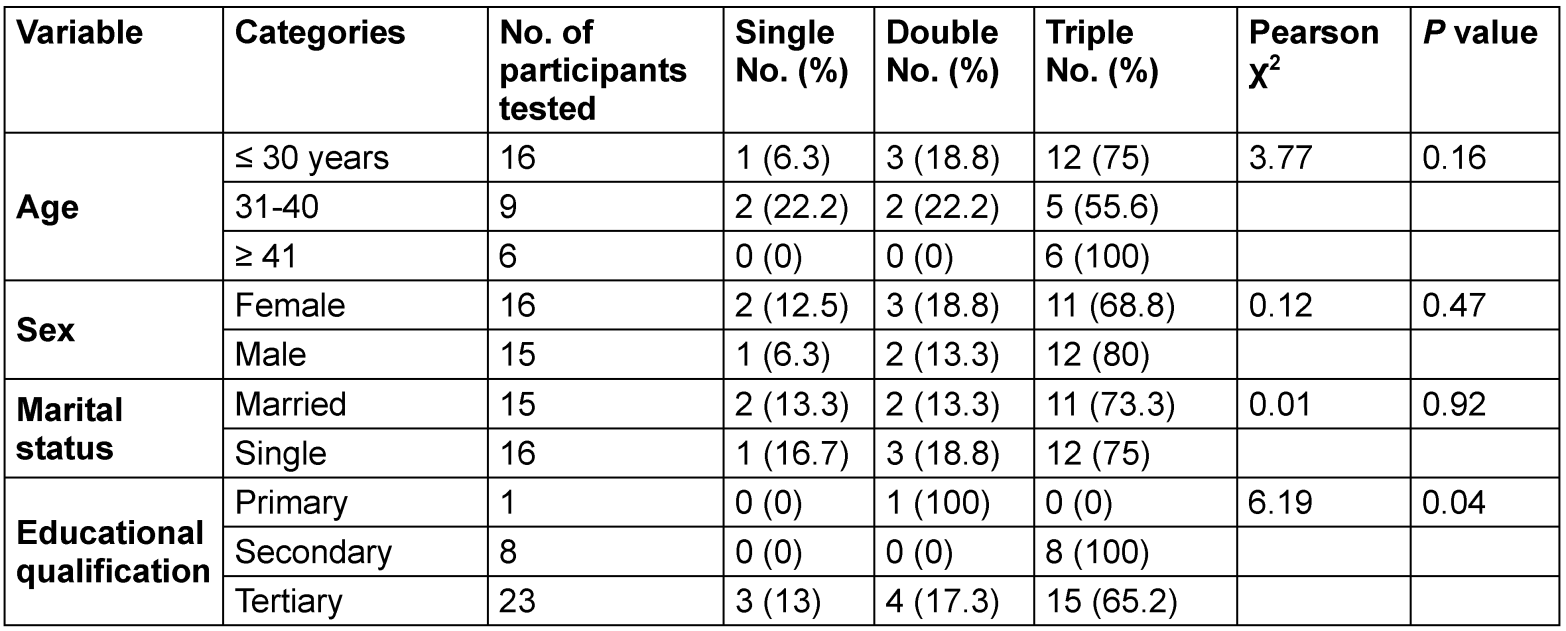
Association between sociodemographic characteristics and triple HBV genotypes
